# Backward spatial perception can be augmented through a novel visual-to-auditory sensory substitution algorithm

**DOI:** 10.1038/s41598-021-88595-9

**Published:** 2021-06-07

**Authors:** Ophir Netzer, Benedetta Heimler, Amir Shur, Tomer Behor, Amir Amedi

**Affiliations:** 1 The Cognitive Science Program, The Hebrew University of Jerusalem, Jerusalem, Israel; 2 The Baruch Ivcher Institute for Brain, Cognition & Technology, The Baruch Ivcher School of Psychology, Interdisciplinary Center Herzliya, Herzeliya, Israel; 3 Department of Medical Neurobiology, Hebrew University of Jerusalem, Hadassah Ein-Kerem, Jerusalem, Israel; 4 Center of Advanced Technologies in Rehabilitation (CATR), Sheba Medical Center, Ramat Gan, Israel

**Keywords:** Sensory processing, Visual system, Human behaviour

## Abstract

Can humans extend and augment their natural perceptions during adulthood? Here, we address this fascinating question by investigating the extent to which it is possible to successfully augment visual spatial perception to include the backward spatial field (a region where humans are naturally blind) via other sensory modalities (i.e., audition). We thus developed a sensory-substitution algorithm, the “Topo-Speech” which conveys identity of objects through language, and their exact locations via vocal-sound manipulations, namely two key features of visual spatial perception. Using two different groups of blindfolded sighted participants, we tested the efficacy of this algorithm to successfully convey location of objects in the forward or backward spatial fields following ~ 10 min of training. Results showed that blindfolded sighted adults successfully used the Topo-Speech to locate objects on a 3 × 3 grid either positioned in front of them (forward condition), or behind their back (backward condition). Crucially, performances in the two conditions were entirely comparable. This suggests that novel spatial sensory information conveyed via our existing sensory systems can be successfully encoded to extend/augment human perceptions. The implications of these results are discussed in relation to spatial perception, sensory augmentation and sensory rehabilitation.

## Introduction

To what extent can sensory perceptions in humans be augmented during adulthood? A wealth of studies addressed this question over the last decades with various approaches. One emerging line of research in this domain explores how novel sensory experiences (NSEs) are perceived by healthy adults. NSEs are sensory information we cannot access through our sensory systems and which humans were not exposed to neither during their lifetime nor during evolution (e.g., perceiving infra-red light). So far, the emergence of NSEs was documented mainly in animals through invasive approaches (e.g., implanting novel sensory receptors in the animals sensory organs) as this appeared to be the only possible way to address this fascinating issue^1–3^.

However, initial evidence suggests that another approach, namely the use of Sensory Substitution Devices (SSDs), might be highly suitable for testing the extent to which perceiving NSEs in humans is even possible^4^. SSDs transform information generally conveyed by one sensory-modality (e.g., vision) into a different one (e.g., audition) using a predetermined algorithm that can be learned by users via dedicated training programs^5–7^. SSDs were originally conceived as rehabilitation tools for the blind and visually impaired populations to convey the missing visual information via intact sensory modalities (audition or touch). By employing SSDs, it was demonstrated that blind users could successfully learn to interpret “visual” information to perform many “visual” tasks via touch or audition such as making judgments of distance^8,9^, recognizing objects^10–13^, perceiving movement^14,15^, navigating in controlled or virtual environments^16–18^ and many more^7,19–21^, including intrinsically visual tasks such as perceiving color^6^ and performing “visual” parsing^22^. However, the intuition at the core of sensory-substitution, namely, the possibility of conveying unperceived sensory information via available sensory inputs using specific sensory transformation algorithms, can be extended beyond visual-impairments. Indeed, it can be re-adapted to convey, in principle, any perceptual information via our existing senses, with the constraints that a suitable transformation algorithm is created, and dedicated training, aiming at learning to interpret the transformed sensory signals, is provided. Encouraging evidence in support of this wide potential of SSDs, comes from previous studies documenting that, for example, healthy adults could learn to perceive information about the direction of the magnetic north, namely, information we do not perceive through our available sensory systems, by wearing a SSD-like belt called the “feelSpace belt”. Specifically, the feelSpace belt delivers continuous vibrotactile stimulations signaling the position of the magnetic north, which changes when people move around in space^4,23^. An additional recent evidence supports this conclusion by showing that novel auditory inputs conveying distance information could be efficiently trained in the adult healthy population, as well as successfully integrated with a noisy visual cue conveying the same information^24^. This latter result suggests that augmented information may facilitate, rather than clash, with existing sensory modalities and related perceptions.

The present study aims at corroborating and expanding this previous evidence^4,23,24^ by focusing on the augmentation of visual spatial perception to include the backspace, namely a region to which humans are naturally blind. Notably, humans do gather some spatial information from this region through audition, though limited to locations of objects that are either moving or that emit a distinct sound (often without recognizing the identity of these objects if the sound they emit is not familiar). To enhance these partial backward spatial representations, we developed an SSD-like algorithm, called the Topographic speech, or in short, the Topo-Speech, which convey spatial “visual” information via the auditory modality (i.e., conveying both identity and locations of objects in the x and y axes, two key perceptual features of spatial vision). Through this algorithm, we aim at conveying more spatially accurate (auditory) backward information, thus potentially enhancing backward topographic representations.

To test the efficacy of this approach, we first examined whether visual spatial information in the frontal visual field, as measured via an objects’ localization task, could be successfully conveyed via the Topo-Speech algorithm in sighted (blindfolded) adults following minimal training (forward condition). Previous available technologies aiming at conveying visual information via audition either provided limited information (e.g., TapTapSee or Seeing AI applications^25–27^ which capture one object at a time and provide its identity through speech, without conveying any spatial information), or required very long training to master (e.g., SSDs which successfully convey both identity and spatial locations of objects, but require long training in order to learn to identify objects shapes^5–7,21,28^). The Topo-Speech was designed to overcome the aforementioned limitations. Specifically, it conveys objects’ identity through speech, similarly to available apps such as TapTapSee, and objects’ locations through sonification procedures, similarly to SSD algorithms (i.e., pitch manipulations represent objects’ positions in the y-axis such that an object that is located in the bottom part of the space will be announced in a low pitch, while an object located on a higher part of the space will be announced in a higher pitch). Positive results will suggest that visual-like spatial information can be successfully learned during adulthood even after very short training, ultimately highlighting the great intuitiveness of this novel algorithm.

Then, and crucially, we investigated, in a different group of sighted (blindfolded) adults, the extent to which the same visual-to-auditory mapping of the Topo-Speech, could be successfully used to convey backward visual spatial information as measured via the same objects’ localization task, tested following minimal training (backward condition). Positive results in this direction will suggest that novel visual-like spatial information can be successfully encoded to extend/augment spatial human perceptions (here for the extension of the human “visual” field to 360°).

Alongside analyzing results in both spatial fields, this design allows the direct comparison between localization performances in forward vs. backward spaces mediated by the Topo-Speech. Previous studies comparing the properties of these two spatial fields, suggest that spatial perception in the frontal field outperform, at least in certain specific tasks, spatial perception in the back at the net of similar auditory sensitivity between the two spatial fields^29–31^ (but see^32,33^). These studies reported worse performance in tasks requiring the building of a precise spatial metric between sounds in the back compared to the frontal spatial field^29–31^. They therefore suggest that vision, and perhaps active motor actions^34^, are both necessary to allow the full development of spatial properties, for instance, by mediating the development of complex spatial field topographies, inclusive of detailed spatial metric representations^29–31^. In line with these studies, one hypothesis is that we will observe better object-localization in the front compared to the back. This would suggest that augmenting backward spatial perception might either require longer training or might be intrinsically impossible due to the lack of visual experience in this portion of space across the lifespan. Alternatively, however, we can hypothesize that performances in forward vs. backward objects’ localization will be comparable. We propose three factors might lead to this outcome: first, the fact that we convey visual-like spatial stimuli in the back, namely mimicking the most accurate spatial modality; second the fact that, before testing, we train users (even if shortly—see “Methods”) on the algorithm while also making them familiarizing with the novel space; third the fact that our users responded through active motor actions (see “Methods”), thus maximizing the familiarization with the novel space^34^. We suggest that these factors might jointly mediate a detailed calibration of the backward space, basically allowing to equate frontal and backward performances. These results would suggest that it is possible, at least to a certain extent, to quickly and efficiently learn in adulthood detailed topographic representations in “blind” areas (namely which lacked vision across the lifespan), and, more generally, to augment human spatial perception via SSD-like algorithms.

## Materials and methods

### Participants

#### Forward condition

Fifteen sighted individuals (nine women; aged 27.2 ± 1.57 years (mean ± SD)) participated in this experiment. All participants were naïve to the Topo-Speech algorithm as well as to any SSDs. Participants were blindfolded across the experimental procedure.

#### Backward condition

A new group of fifteen sighted individuals (eight women; aged 27.07 ± 4.17 years (mean ± SD)), participated in this experiment. All participants were naïve to the Topo-Speech algorithm as well as to any SSDs. Participants were blindfolded across the experimental procedure.

This experiment was approved by the institutional review board of the Interdisciplinary center (IDC) Herzliya. All participants signed an informed consent form before starting the experiment and received a monetary compensation for their participation in the study. Informed consent was also obtained for publication of identifying information/images in an online open-access publication. All research was performed in accordance with the relevant guidelines and regulations.

### Equipment

#### The Topo-Speech algorithm

**Figure 1:**
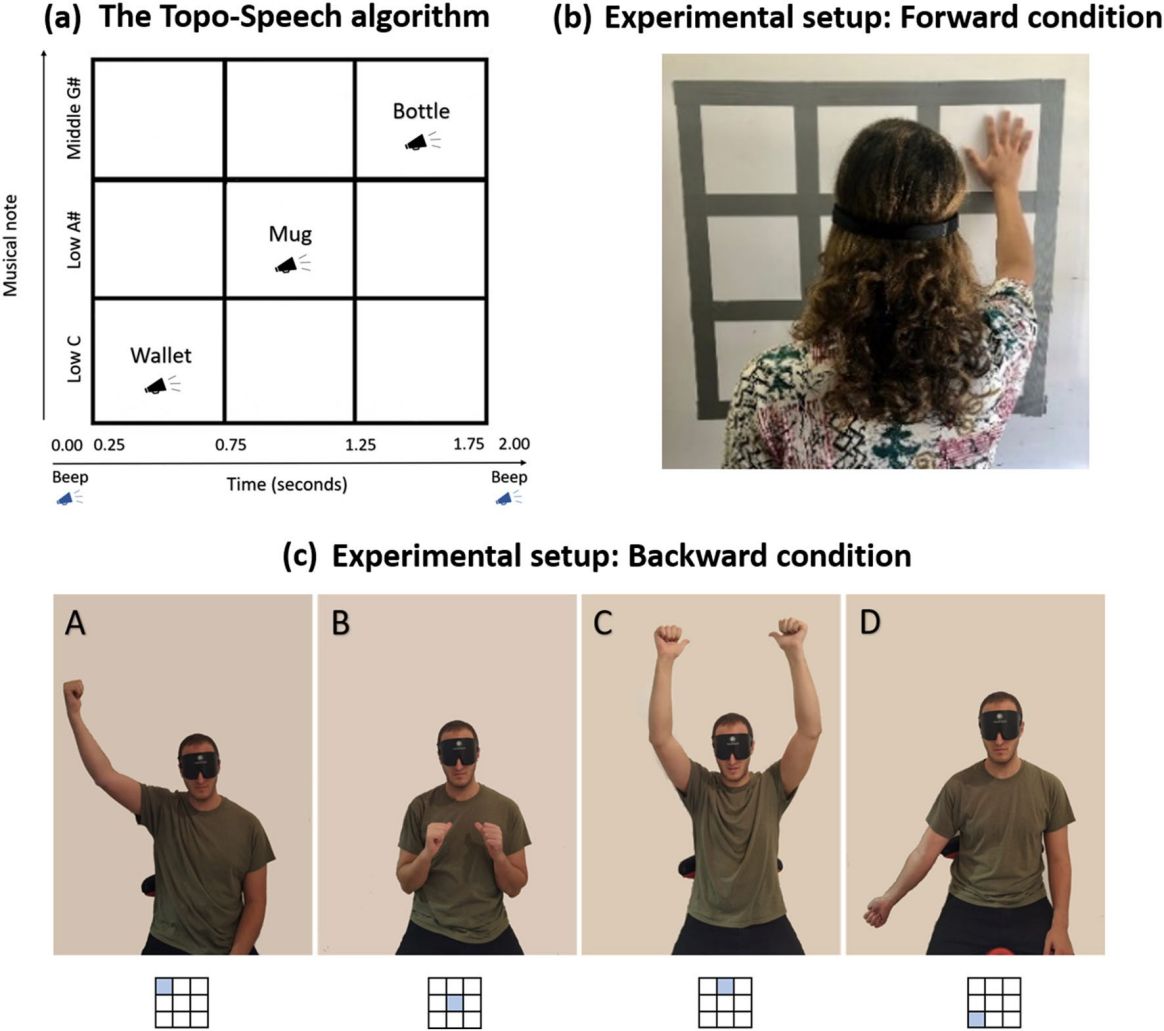
(**a**) Illustration of the Topo-Speech algorithm and of the temporal structure of a trial. (**b**) Illustration of experimental setup in the forward condition. (**c**) Illustration of experimental setup in the backward condition with some of participants’ pointing gestures which they used to provide responses. A—The participant is pointing back to the top right corner of the 3 × 3 grid. B—The participant is pointing back to the central location of the 3 × 3 grid (the middle of both the y-axis and x-axis). C—The participant is pointing back to the top-middle location in the 3 × 3 grid. D—The participant is pointing back to the bottom right corner of the 3 × 3 grid.

The Topo-Speech algorithm combines an SSD-like algorithm with speech information. Specifically, this algorithm conveys the spatial arrangement of a given image both in the x and y axes (Fig. 1a). The algorithm scans an image from left to right with a sweep-line approach. Thus, the x-axis is mapped to the time domain: objects located on the left side of the image will be heard before objects located on the right side of the image. In addition, the beginning of the scan is signified by a “beep” sound (far left), then the algorithm scans through the entire image, and the end of the scan is signified by another, different “beep” sound (far right). The two beeps are easily distinguishable, and so they define the borders of the image in the left–right dimension. The y-axis is mapped to the frequency domain (pitch) using the pentatonic scale: the higher the object is located, the higher in pitch the musical note sonifying it will be. Objects’ identities are conveyed through language.

Accordingly, the higher in pitch a word would sound, the higher in space the corresponding object would be. In addition, the closer to the first beep the word would sound, the more the corresponding object would be located to the left side of the space.

Specifically, we aimed here to create a 3 × 3 grid of possible objects locations: for conveying the objects’ positions in the y-axis we recorded words with the help of a professional singer to be played in one of three different pitches: low C (i.e., bottom y-axis position), low A# (i.e., central y-axis position), middle G# (i.e., top y-axis position) (Fig. 1a).

For conveying objects’ positions in the x-axis, we set the length of each word to 50 ms and created a specific timeline during each trial (Fig. 1a). Each trial lasted 2 s and was divided as follows: a delay of 5 ms between the onset of the trial and the initial beep; the initial beep lasting 10 ms; a delay between the initial beep and the first (left) possible stimulus position (10 ms); one of the words for 50 ms (left: between 25 and 75 ms from the onset of the trial; central: between 75 and 125 ms from the onset of the trial; right: between 125 and 175 ms from the onset of the trial); a delay between the last (right) possible stimulus position and the final beep (10 ms); the final beep (10 ms) and a delay between this beep and the end of the trial (5 ms). This structure was kept constant for all stimuli (Fig. 1a).

The stimuli and the Topo-Speech algorithm used were identical in both tasks (forward and backward conditions). The experiment was programmed using OpenSesame. In both conditions, participants provided their responses via motor actions (specifics are provided in the experimental procedure of each condition), and the experimenter entered them to the computer program.

#### Experimental stimuli

Stimuli consisted of a pool of 60 Hebrew words: all words were highly frequent in the Hebrew language, two syllables long and were all graspable objects. All words were recorded by a professional singer and then further modified with the Audacity software to fit the requirements of the Topo-Speech algorithm and of the structure of the trials (see details above).

#### Word selection

In order to exclude from the experimental stimuli pool words with spatial bias in the y-axis (high vs. low) that might influence participants’ responses (e.g., “shoe”-may be associated with the lower part of the space; “hat”-may be associated with the upper part of the space), we performed a spatial bias judgment survey on a group of 11 participants (six women, aged 30.09 ± 11.87 years mean ± SD). Specifically, in each trial participants were presented with one of the originally selected 60 words appearing in its written form on the center of a computer screen, and under the word a continuous bar would appear with a movable block positioned in the middle of the bar (see Supplementary Fig. S1). Their task was to read each word and decide if the they felt the word had a spatial bias, in which case they were instructed to use the mouse to move the block of the bar toward the right (low spatial bias) or toward the left (high spatial bias) according to the strength of such perceived bias. Twenty-six out of the total of sixty words were found to be associated with a significant spatial bias in one of the two directions (either high or low—see “Supplementary material” for full analyses and see Supplementary Table S1 for full list of words). These words were removed from the pool of words used for the Topo-Speech experiment. Thus, for the experiment we selected a pool of 30 words which had no spatial bias regarding spatial position (e.g., “sargel”-ruler; “matzit”-lighter). For the short training phase on the Topo-Speech algorithm, we used a different pool of 20 words to avoid any memory effect which might have an influence on responses in the actual experiment. Thus, 16 of the 20 words used during training, were selected among the words which resulted significantly associated with a spatial bias in one of the two directions.

### Experimental set-up

#### Forward condition

A 3 × 3 grid was created on a whiteboard hanging on a wall, (80 × 80 cm) which was divided into three rows and three columns, creating nine inner cells. The outline of the grid was created using a textured duct tape, so that participants could discriminate between cells through touch alone while blindfolded (Fig. 1b).

#### Backward condition

The experimental set-up was identical to the one described for the forward condition, though in this condition participants were tasked to localize stimuli appearing behind the back of their body: what we term backward vision (i.e., extending our visual field to include visual information presented behind our backs, thus normally unavailable to our visual system, using the Topo-Speech algorithm). During the entire experimental procedure, participants were seated on a chair with their back against a wall (differently from the forward condition where participants were seated facing a whiteboard) (Fig. 1c). In addition, during this task participants were asked to point at the cell location where they thought the Topo-Speech stimulus was located (Fig. 1c; see “Experimental procedure” for further details), rather than touching it, as was required in the forward condition. This latter change was introduced due to the limited ability of touching the board to identify locations behind the back. Therefore, there was no need to create the 3 × 3 grid with duct tape that we created for the forward condition (Fig. 1c).

### Experimental procedure

#### Forward condition

The experiment was composed of two parts: a short training session followed by the experiment. For both parts, participants sat in front of the whiteboard and were instructed to first hear the stimuli conveyed by the Topo-Speech and then touch the cell on the board in which they thought each stimulus was located. The experimenter entered their responses into the computer. Participants heard in each trial one single object (i.e., one single word). Before starting the training, the experimenter briefly explained to the participants the concept of SSD and the basic principles of the Topo-Speech algorithm: “SSDs are algorithms that convey visual information via other sensory modalities, in this case-audition. The Topo-Speech algorithm maps a space of three rows and three columns, creating a 3 × 3 grid conveying the identity of objects via speech and their spatial locations according to these rules: the x-axis is mapped to the time domain from left-to-right, so the sooner you hear a stimulus the more it will be left localized; The y-axis is mapped to the frequency domain, so the higher in pitch you’ll hear a word, the higher on the grid the corresponding object will be localized. Then, participants were told that in each trial they will hear only one word and that the beginning and the end of each word presentation, representing the beginning and the end of x-axis space, were signaled by two different beeps, of which they heard an example. Finally, participants were asked to sit in front of the whiteboard to start the training and get familiar with the Topo-Speech stimuli for the first time. During training, trials were presented in a random order, with two constraints: each of the nine possible locations had to be tested three times, but words could not appear twice in the same location. The training contained 27 trials in total. Participants were informed that they could hear each word in each trial as many times as they wanted. The experimenter gave feedback on participants responses and directions to help participants study their mistakes in order to better learn the algorithm. Every time participants gave an incorrect response, the experimenter would inform the participants and the program would play the trial again until a correct response was provided, before moving to the next trial. The duration of the training circled around 10 min.

At the end of training, participants proceeded to the experiment. During the actual experiment, participants were informed that the task would remain the same as during training, but they could hear each word only twice before providing a response, and no feedback was going to be provided by the experimenter. Participants were encouraged to respond as quickly and as accurately as possible. Words appeared in each location ten times, creating 90 trials in total. Similar to the training, each word could be repeated during the experiment, but it could not appear again at the same location to make sure there was no link between specific words and specific locations. The experiment lasted around 20 min, including a short break in the middle.

#### Backward condition

Participants sat with their backs to a wall and were instructed to use hand gestures pointing back to indicate the location of the object appearing on a 3 × 3 grid behind them (Fig. 1c). The hand gestures were practiced before the experiment to make sure participants were able to use them correctly. All other parts of the experimental design and procedure were identical to the forward condition.

### Statistical analyses

For all statistical analyses performed in both forward and backward conditions, we used non-parametric tests, even though the null hypothesis in a Shapiro–Wilk test was not rejected (all p-values > 0.52). This was decided upon considering two properties of our data, both limiting the reliability of parametric analyses if applied: (A) the sample size in each condition is relatively small (< 30 in all analyses performed); (B) an evaluation of the density plots for the data in each condition separately, showed that the evaluated distributions tended to both be negatively skewed (see Supplementary Fig. S2). Specifically, we used the Wilcoxon signed-rank test for paired comparisons, the Wilcoxon rank sum test (equivalent to the Mann–Whitney test) for independent-samples comparisons, the Kruskal–Wallis test for repeated measures variables or the Friedman test of differences for between-group comparisons. Post-hoc tests were conducted using the Bonferroni correction. Additionally, to test the equivalence of the forward and backward performance distributions, we used non-parametric equivalence testing^35,36^, in the form of two one-sided Wilcoxon tests^35^. Equivalence tests allow us to statistically reject effects large enough to be deemed worthwhile, even if no statistically significant difference was found^37,38^. Equivalence was established if the 95% confidence interval (CI) of the median difference between the forward and backward conditions fell within the equivalence boundaries. The equivalence boundaries were set to one standard error (SE) of the forward condition distribution (± 0.024% points), namely a strict criterion to be able to exclude the existence of even a slight tendency for better accuracy in the forward condition compared to the backward condition (e.g., as was shown in previous studies^29,31^). The equivalence test was performed on the difference distribution ($\Delta )$ of success rates between the forward and backward conditions. The two one-sample Wilcoxon tests were performed against the equivalence boundaries of ${\Delta }_{U}=0.024$ and ${\Delta }_{L}=-0.024$.

In both forward and backward conditions, one participant from each group was identified as an outlier. In both cases, the participant’s average performance was more than 2 standard deviations below the overall group mean, and therefore both participants were excluded from the pool of analyzed subjects. Thus, all analyses were computed on 14 participants in each condition. We will now present results separately for the forward and the backward conditions as well as performance comparisons between the two conditions.

## Results

### Training

**Figure 2:**
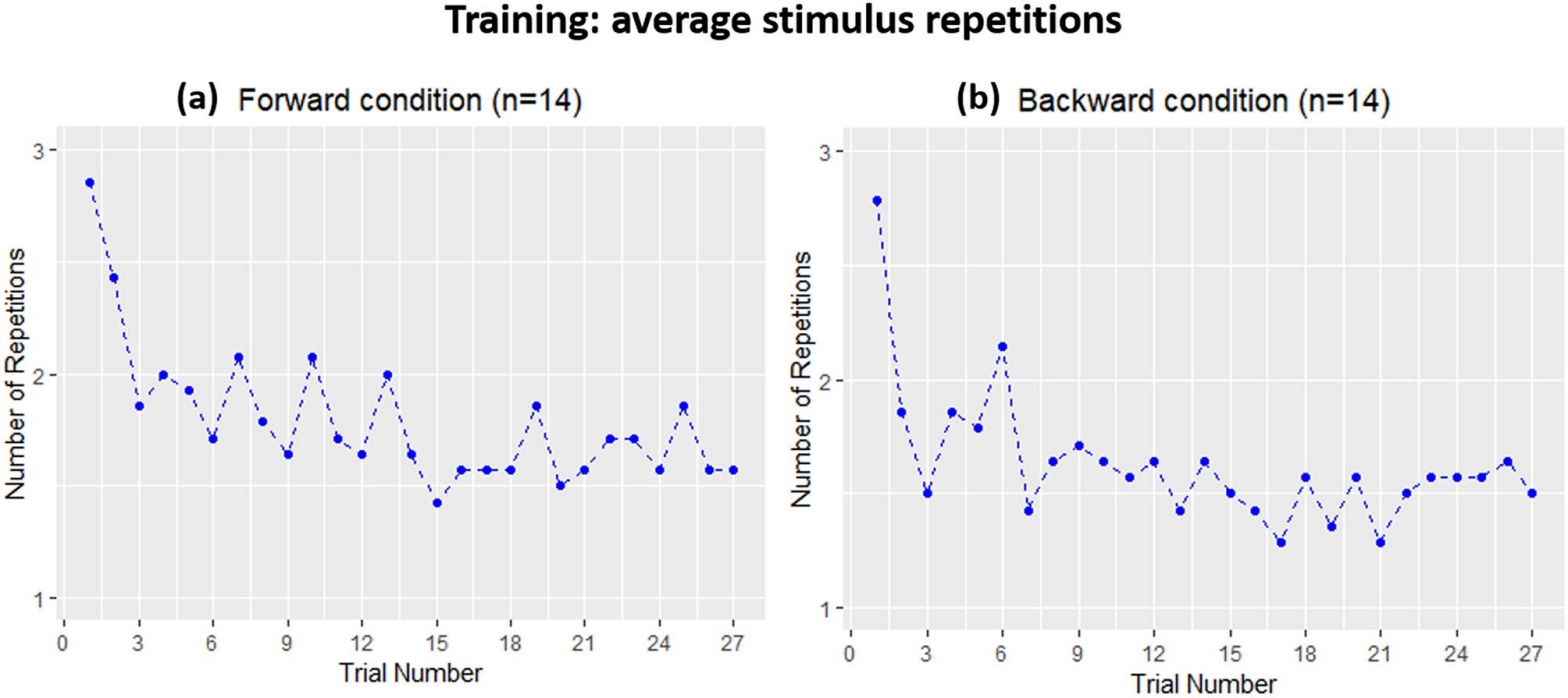
Average number of stimulus repetitions before providing the correct response as a function of trial number during training (~ 10 min). (**a**) Blindfolded sighted participants in the forward vision condition (n = 14). (**b**) Blindfolded sighted participants in the backward vision condition (n = 14). During the short training, the average number of repetitions before providing the correct response decreased across trials in both conditions, suggesting that the training was effective in teaching to correctly interpret the algorithm. This Figure was created using R Core Team (2018). R: A language and environment for statistical computing. R Foundation for Statistical Computing, Vienna, Austria. R version 3.5.2 (2018-12-20). https://www.R-project.org/.

To evaluate the effectiveness of the training program for teaching participants the Topo-Speech algorithm, we calculated for each trial the average number of trial repetitions before participants provided the correct response (during training participants could hear each stimulus as many times as they wanted and by default heard stimuli again if they gave an incorrect response). Thus, the lower the number of repetitions across trials, the better was the learning of the algorithm. Figure 2 demonstrates that average stimulus repetitions diminished during training for both the forward (Fig. 2a) and the backward conditions (Fig. 2b).

### Experimental task

After completing the training, participants moved to the experiment. Table 1 specifies the overall success rate and standard deviation (SD) for each participant in the forward condition. The group average and SD are: $80.24\%\pm 9.08\%$. The overall performance of participants was greater than chance (Median (Mdn) = 80.56%, Z = − 3.27, p-value < 0.0006; one-sample one-tailed Wilcoxon signed-rank test).

**Table 1:** Individual success rates (and standard deviations (SD)) in blindfolded sighted participants during the forward object-localization task.

Success rate in the 3 × 3 object localization: forward condition		
Subject number	Success rate (%)	SD (%)
1	83.33	37.48
2	93.33	25.08
3	74.44	43.86
4	91.11	28.62
5	74.44	43.86
6	71.11	45.58
7	77.78	41.81
8	75.56	43.22
9	86.67	34.18
10	84.44	36.45
11	84.44	36.45
12	61.11	49.02
13	74.44	43.86
14	91.11	28.62

Table 2 specifies the overall success rate and standard deviation (SD) for each participant in the backward condition. The group average and SD are: $79.60\%\pm 8.65\%$. The overall performance of participants differed from chance (Mdn = 79.44%, Z = − 3.27, p-value < 0.0006; one-sample one-tailed Wilcoxon signed-rank test).

**Table 2:** Individual success rates (and standard deviations (SD)) in blindfolded sighted participants for the backward object localization task.

Success rate in the 3 × 3 object-localization: backward condition		
Subject number	Success rate (%)	SD (%)
1	75.56	43.22
2	92.22	26.93
3	74.44	43.86
4	87.78	32.94
5	83.33	37.48
6	78.89	41.04
7	91.11	28.62
8	77.78	41.81
9	80.00	50.22
10	58.89	49.48
11	72.22	45.04
12	82.22	38.45
13	74.44	43.86
14	85.56	35.35

**Figure 3:**
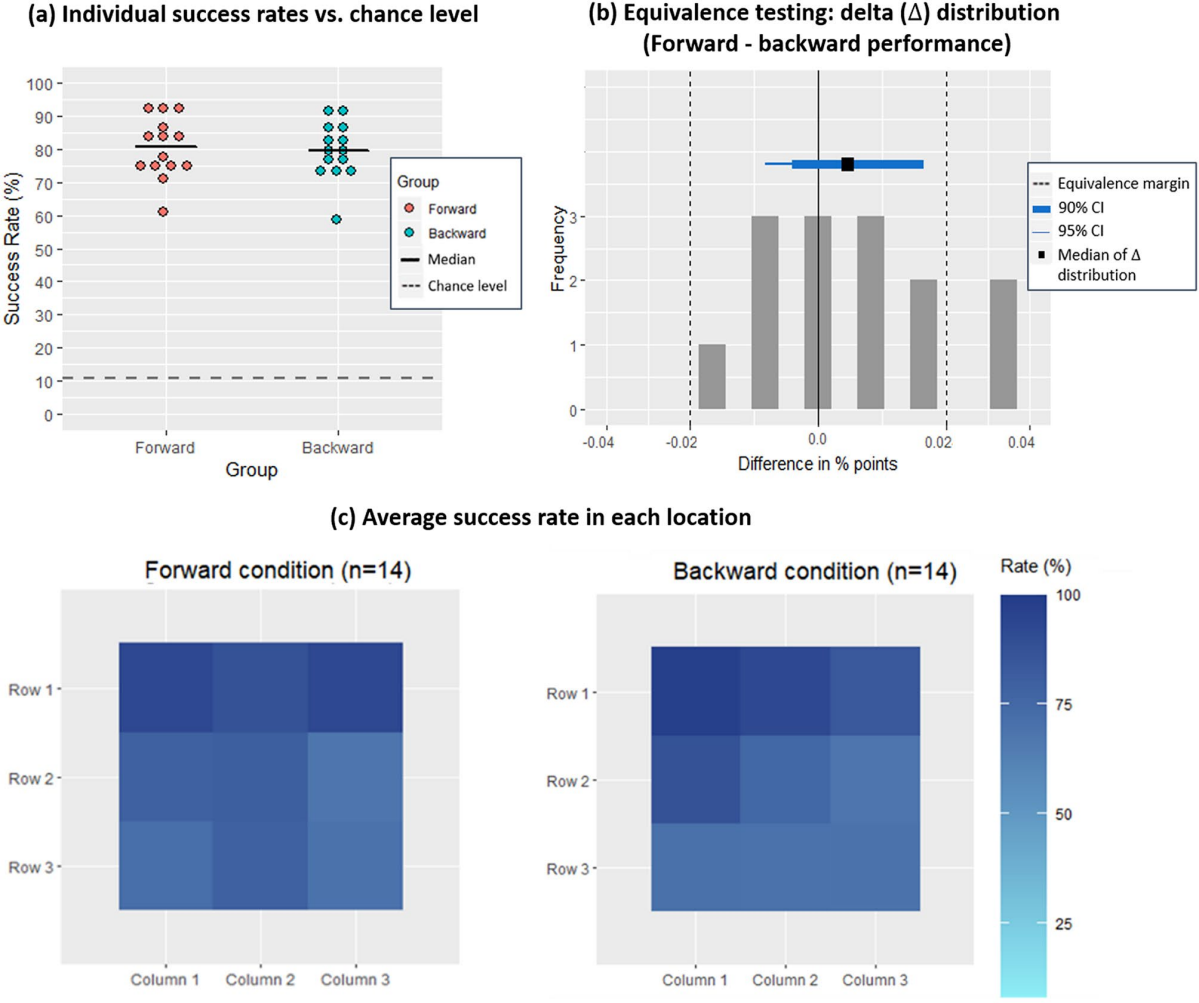
(**a**) Individual average success rates in the forward vs. backward conditions: individual average success rates plotted against chance level (11%) in forward (pink) and backward (turquoise) conditions. Each colored dot represents the average success rate of one participant. Chance level is represented by the dashed gray line. All participants in both groups performed well above the chance level. The median of each group is represented by a solid black line. The two individual accuracy distributions closely resemble each other, further suggesting entirely comparable performance in the forward vs. backward fields. (**b**) Equivalence testing using the delta ($\Delta$) distribution (forward–backward performance). The grey columns represent the frequency of values of the delta distribution (i.e., the difference in percentage points between forward and backward performances). The dashed black lines represent the upper and lower equivalence bounds ${(\Delta }_{\mathrm{L}}=-0.024$ and ${\Delta }_{\mathrm{U}}=0.024)$. The thick blue line represents the 90% confidence interval (CI) of the $\Delta$ distribution [− 0.005, 0.02]. The thin blue line represents the 95% CI of the $\Delta$ distribution [− 0.01, 0.02]. The black square represents the median of the $\Delta$ distribution (M = 0.0055). As we can observe, both the 90% and the 95% CIs include the value of 0, but do not include the equivalence boundaries. Thus, we can conclude that the difference between success rates distributions of the forward and backward conditions is not statistically significant and the distributions are statistically equivalent. (**c**) Heat maps for the average success rate in each of the nine cells across participants. Bottom left: blindfolded sighted participants in the forward condition (n = 14). Bottom right: blindfolded sighted participants in the backward condition (n = 14). In both panels, each cell on the 3 × 3 grid is color-coded based on the average success rate in it. Darker shades of blue represent higher success rates. Accuracy was very high for both groups of participants in all cells. This Figure was created using R Core Team (2018). R: A language and environment for statistical computing. R Foundation for Statistical Computing, Vienna, Austria. R version 3.5.2 (2018-12-20). https://www.R-project.org/.

We compared the performance between the forward and backward conditions using an unpaired Wilcoxon rank sum test and results showed no significant difference (Z = 1.45, p-value = 0.9265), thus suggesting that participants performed comparably well when using the Topo-Speech algorithm to localize objects in the forward or in the “backward” visual field (Fig. 3a). Figure 3a, which reports the individual average success rates plotted separately for each condition, reinforces this statistical analysis results. Indeed, it shows that (a) all participants performed well above chance level (11%; gray dashed line); (b) the two individual accuracy distributions closely resemble each other; and (c) the medians of each condition are strikingly similar, also suggesting the similarities between the two distributions.

To further investigate potential differences between the forward and backward performance distributions, we performed an equivalence non-parametric test in the form of two one-side Wilcoxon tests. These tests were performed against the equivalence boundaries of ${\Delta }_{U}=0.024$ and ${\Delta }_{L}=-0.024$ and revealed that performance in the two conditions was statistically equivalent (all p-values < 0.0023, ${Z}_{U}=-2.83, {Z}_{L}=-3.27$; Fig. 3b). As shown in Fig. 3b, both the 90% and 95% confidence intervals (CI 95% CI [− 0.01, 0.02]; 90% CI [− 0.005, 0.02]) calculated on the delta ($\Delta )$ distribution of the median differences in accuracy between forward and backward conditions (median of $\Delta \mathrm{distribution}:0.0055),$ do include the zero value, namely confirming the difference between the two distributions is not statistically significant. In addition, both CIs lie within the equivalence boundaries thus confirming the two distributions are also equivalent^38^ (Fig. 3b).

In addition, we were interested in investigating whether one of the two dimensions (pitch to map the y-axis vs. time to map the x-axis) was harder to learn/perceive compared to the other, as well as whether certain locations (specific cells in the 3 × 3 grid) were easier or harder to identify. To test for a possible difference in learning between the two dimensions of the Topo-Speech algorithm, we performed a paired-samples Wilcoxon signed rank test between the success rate of each participant in the y-axis (locating the correct row of the object, independently of the column) and the success rate of each participant in the x-axis (locating the correct column of the object, independently of the row). In the forward condition, this analysis revealed no significant difference in participants’ performance between rows and columns (Z = 0.08, p-value = 0.53; rows overall accuracy Mdn: $90.56\%$, columns overall accuracy Mdn: $88.89\%$). Similarly, this analysis revealed no significant difference in the performance of participants in the backward condition between rows and columns (Z = 0.53, p-value = 0.7; rows overall accuracy Mdn: $88.89\%$, columns overall accuracy Mdn: $91.11\%)$. Moreover, no significant difference between performances in the two dimensions was observed when comparing results from the forward and backward conditions, neither in the x-axis (Z = − 0.84, p-value = 0.80) nor in the y-axis (Z = − 0.17, p-value = 0.43) using an unpaired-samples Wilcoxon signed-rank test.

To investigate possible differences in success rates in each of the nine locations of the 3 × 3 grid, we performed a Kruskal–Wallis test using the average accuracy of each cell as the dependent variable. In the forward condition, a statistically significant difference among locations was found (Chi-squared = 38.75, p-value < 0.0001, df = 8). Post-hoc tests using the Bonferroni correction revealed a difference in success rate between the upper-left grid cell (row 1, column 1) and the middle-right grid cell (row 2, column 3; p-value < 0.04) and between the upper-right grid cell (row 1, column 3) and the middle-right grid cell (row 2, column 3; p-value < 0.04). In the backward condition, a statistically significant difference was found between locations (Chi-squared = 43.88, p-value < 0.0001, df = 8). However, differences in accuracy among locations did not survive the Bonferroni correction. Importantly, when comparing the average success rate for each possible location between the two conditions, no significant difference emerged using the Friedman test (Chi-squared = 0 p-value = 1, df = 1). The heat maps in Fig. 3c depict average accuracy in each of the nine cells. When observing the heat maps of the two conditions one can notice that participants in both conditions tended to identify better on average objects located in the top row than objects in other locations, though, in both conditions, accuracy was very high in all locations.

**Figure 4:**
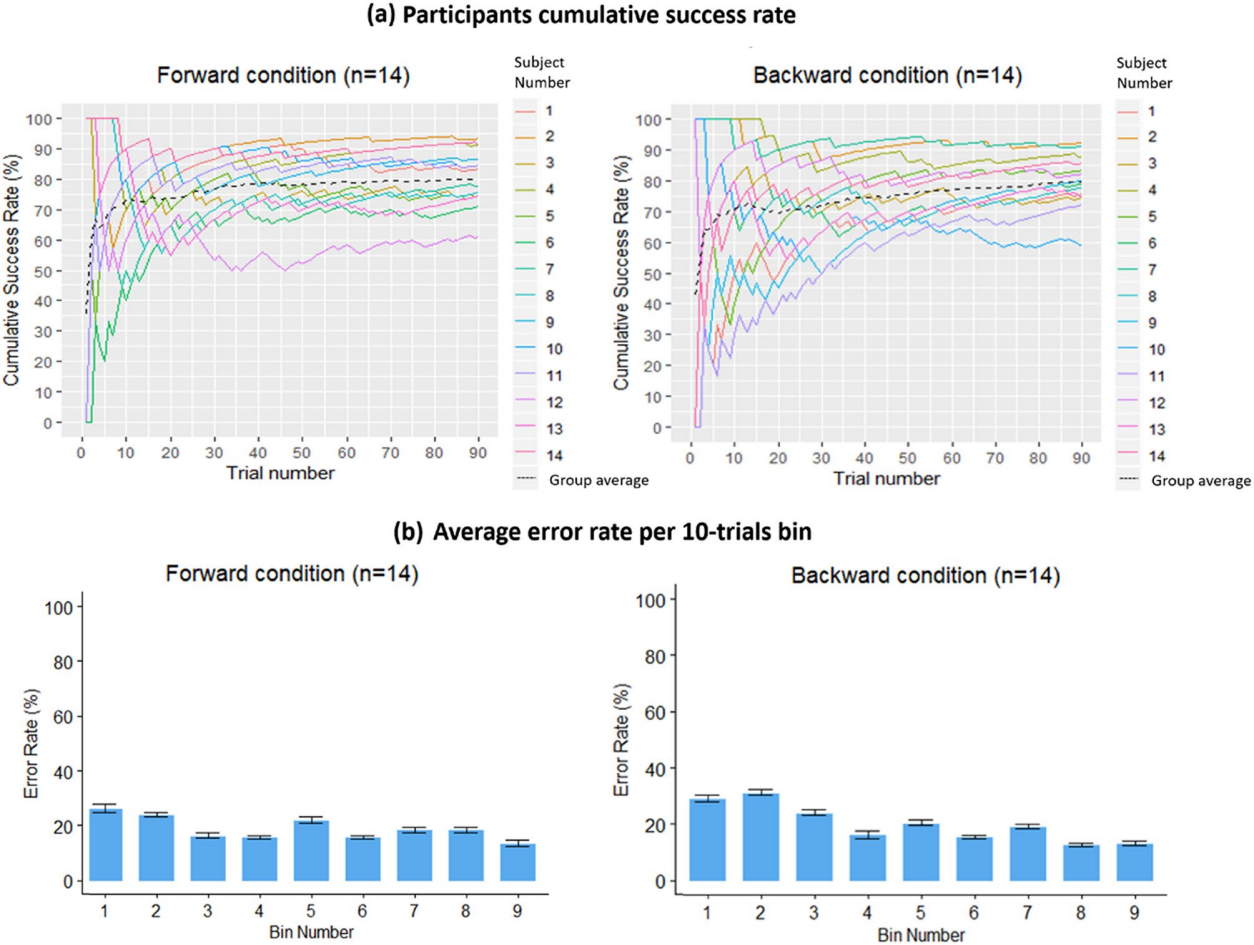
(**a**) Cumulative success rate for each participant as a function of trial number. Upper left: Blindfolded sighted participants in the forward condition (n = 14). Upper right: Blindfolded sighted participants in the backward condition (n = 14). In both panels, cumulative success rate for each subject is represented by a different colored line, while the group average is represented by the dashed black line. Both groups show a steep increase in accuracy within the first ten trials, after which all participants reach a plateau, maintaining a relatively stable performances for the rest of the experiment. (**b**) Average error rate as a function of the bin number across the experiment. Bottom left: Blindfolded sighted participants in the forward condition (n = 14). Bottom right: Blindfolded sighted participants in the backward condition (n = 14). Each bin represents the average error rate for every 10 trials, and so there are 9 bins representing the course of the 90 trials presented in the experiment. Error bars depict standard deviations from the mean. A tendency for diminishing mistakes during the experiment can be observed, especially for the backward condition. This Figure was created using R Core Team (2018). R: A language and environment for statistical computing. R Foundation for Statistical Computing, Vienna, Austria. R version 3.5.2 (2018-12-20). https://www.R-project.org/.

In addition to the success rate, given the short training on the Topo-Speech (~ 10 min) at the beginning of the experimental procedure, we were interested in investigating whether participants continued learning during the experiment as well as the properties of such ongoing learning. To this aim, we modeled the cumulative success rate for each participant during the experiment, i.e., we calculated for each trial the correct responses given thus far by each participant divided by the number of trials presented. Figure 4a presents the individual results separately for each group in each condition together with the group average cumulative success rate: in both groups, participants steeply improved in the first ten trials. After which they reached a plateau, though maintaining a high steady cumulative success rate till the end of the experiment (i.e., a stable and high ratio between correct and incorrect responses). This suggests a similar pattern of learning between the groups in the forward and backward conditions.

Another approach we took to assess participants’ learning during the experiment was to model the error rate of participants across trials. We divided the experiment into bins of ten trials, creating nine bins. For each 10-trials bin we calculated the average percentage of errors made across participants. Figure 4b presents the results. We can see a tendency for a decrease in the average error rate of participants during the experiment in both groups, with a higher error rate in earlier compared to later bins of trials (Fig. 4b). This may suggest that participants self-calibrated and continued to learn during the experiment without any feedback from the experimenter. In the forward condition, the average error rate did not significantly differ among bins as determined by Kruskal–Wallis tests (Chi-squared = 10.53, p-value = 0.23, df = 8). In the backward condition, learning across bins seemed to be confirmed by the results of the Kruskal–Wallis test, which showed a significant difference in the average error rate among bins (Chi-squared = 27.54, p-value < 0.0006, df = 8). However, no significant difference emerged between specific bins after the post-hoc Bonferroni correction was applied (all p-values > 0.31). Note that the error rate is small across all bins, confirming that participants learned the algorithm well and were successful in using it. Finally, no significant difference in the average number of mistakes in each of the nine bins emerged when comparing results between the two conditions using the Friedman test (Chi-squared = 0.5 p-value = 0.48, df = 1).

## Discussion

In this work, we evaluated the extent to which we could successfully augment visual spatial perception via audition, to include the backward space, namely an area to which humans are naturally blind. Specifically, we developed a novel SSD-like algorithm, the Topo-Speech, which convey auditorily both identity and exact spatial locations of objects (i.e., two distinctive features of visual spatial perception) to both the forward and backward spatial fields. Our main aim was to assess the intuitiveness of this algorithm and compare its efficacy between the aforementioned two spatial fields to ultimately investigate the properties of backward spatial augmentation. Results showed that blindfolded participants, entirely naïve to the Topo-Speech algorithm and to the concept of SSDs, were able to locate objects positioned on a 3 × 3 grid either in front of them (forward condition) or behind their backs (backward condition) with comparable high level of accuracy, after only ~ 10 min of training. These results carry implications on various domains spanning from basic research to rehabilitation, which we will address in greater detail below.

### Extending visual spatial perception

The current results suggest that it is possible to extend typical properties of visual spatial perception (i.e., successfully conveying together both the location and the identity of objects) to the backward space, namely, an area of the environment that humans cannot normally explore through vision. In particular, the present results highlight that accuracy in the forward condition (in which blindfolded participants were asked to locate objects in the frontal field of view using the Topo-Speech algorithm) and in the backward condition did not differ (see Fig. 3). This suggests that the Topo-Speech algorithm was equally efficient in conveying via audition both types of information: the natural, yet sensory-substituted ability (auditory forward localization), as well as the extended ability (auditory backward localization). Note that two separate groups of participants performed the two spatial conditions, thus excluding that any training effect might have influenced the results.

We refer to auditory backward localization as the extended ability because humans can only very moderately explore this spatial area through audition. Specifically, locating objects in this area can be done only for objects that emit sounds, and their identity can be decoded only if the sound is familiar. Various solutions that have been previously developed attempted to enhance the monitoring of the backward space by increasing backward localization abilities through sonification strategies. This is the case, for instance, of distance sonification systems in cars. Even though users do find these systems useful, they provide more limited backward spatial information compared to the Topo-Speech algorithm. First, available systems usually convey spatial information regarding the horizontal plane only, leaving out the sometimes-crucial information regarding the vertical dimension. In addition, these systems do not convey object identity information which is essential for a complete exploration and analysis of the environment (e.g., is the object behind me a trash can, a person, or a moving car?). The Topo-Speech algorithm overcomes these limitations, which, as suggested by the current results, can potentially allow the creation of a more precise backward topographic map (Fig. 3c).

Importantly, the current results also differ from, and potentially extend, conclusions reached by previous studies investigating differences between spatial performances in the front and in the back^29,31^. These studies systematically reported worse spatial performances in the back compared to the frontal field at the net of equal auditory sensitivity in the two spatial fields^29,31^. The authors explain the results within the framework of the sensory calibration theory, which states that the most accurate sense to carry out a specific computation teaches (calibrates) the others, thus enabling them to also successfully carry out that same specific computation; however, when the calibrating modality is missing, that specific computation would be impaired when carried out via other sensory modalities^39–43^. The authors argued that since vision is the most accurate sense to carry out spatial processing, when missing (such as in the case of the back of the space), spatial processing in the visually-deprived region will be impaired^29,31^. In particular, Aggius-Vella et al. argued that the lack of visual calibration in the backward spatial field might determine their reported lower backward spatial processing accuracy, for instance by preventing the proper development of fine topographic maps and related detailed spatial metric representations concerning this portion of space^29,31^. Interestingly, beside reporting different conclusions from the current results, the aforementioned studies and our current work used a similar experimental task. Specifically, Aggius-Vella et al. used a spatial bisection task during which participants heard three consecutive sounds on the same horizontal plane, and needed to report verbally if the second sound they heard was closer in space to the first or to the third sound^29,31^. Our Topo-Speech localization task was rather similar, as also in our task participants heard three consecutive sounds: a beep-sound signaling the beginning of a trial, the stimulus (of which the position varied in a 3 × 3 possible grid of options), followed by another, different beep-sound signaling the end of the trial (Fig. 1a). And indeed, for the x-axis localization, the task of the participants in our experiment was essentially similar, namely deciding in which of the three horizontal cells the Topo-Speech stimulus was located, based on its relative distance from the beginning and the end beep-sounds.

The differences in outcomes between the current study and the aforementioned studies^29,31^ can be explained by several factors which might have facilitated the current task: first, in the current study we provided sounds localization information in the vertical plane as well as in the horizontal plane. This might have facilitated correct localizations by providing multiple cues to users to identify the exact spatial position. Note that this is also a more ecological experience, resembling more closely localization in real life environments. Second, we used auditory stimuli that included both identity and location information, rather than using simple and meaningless sounds. We chose these stimuli because their properties more closely resemble visual spatial properties, and thus might mediate a finer calibration of this novel space. Third, we trained our participants on the algorithm, albeit shortly, thus allowing them to familiarize with the new backward space before testing their performance (see^44^ for results confirming that training can indeed facilitate backward spatial perception). Fourth, we provided participants with feedback during training, which informed participants about the relative reliability of sensory cues, ultimately possibly facilitating sensory learning^24,45^. Finally, we asked participants to respond through actions rather than verbally. Establishing sensorimotor contingencies between the percept and the external world (i.e., learning the systematic relations occurring between actions and sensory information), is a process considered crucial for the rise of perceptual awareness^34,46,47^. Thus, answering through actions, might have, in turn, improved/facilitated the learning of the novel spatial cues.

Future studies should shed further light on these issues and identify which factor or combination of factors is responsible for the elimination of the previously documented disadvantage for the back compared to the frontal space^29,31^. Another option, however, is that the current localization task was simply not challenging enough. In this study we tested three possible x-axis positions, while the previous aforementioned studies tested as much as eleven x-axis locations^29,31^. It might be then, that by making localization on the horizontal plane more difficult by adding more possible locations, the back performance will deteriorate (see section below on the limitations of the current study). Nonetheless, these results suggest the intriguing conclusion that at least to a certain extent, it is possible to augment visual spatial properties through audition to include the backward space, a naturally-blind region of space. Future studies should investigate more thoroughly the properties and the possible constraints of such augmentation and should better define the role of both vision and action experiences in shaping such properties and related constraints.

### Conveying novel sensory experiences (NSEs) to healthy adults via sensory-substitution

Taken together, the current results seem to suggest that it is indeed possible to convey novel sensory experiences (NSEs) to healthy adults via the existing sensory systems. Specifically, our findings extend previous initial results suggesting that healthy adults can successfully augment their spatial perception via the existing sensory systems, using sensory transformation algorithms^4,23,24,47^. For instance, the König lab designed a sensory augmentation device, called the feelSpace belt^48^ which is a belt that when worn, continuously tracked the position of the magnetic north during participants’ free movement and conveyed it via vibrotactile stimulation around the waist, i.e., the position of the magnetic north changes in relation to the body when people move around, but humans cannot constantly perceive it with their available sensory systems^4^. Thus, the feelSpace belt provides directional information for which humans do not have a natural perception (as opposed to many animals)^47^. In a subsequent study, the group reported that training led to changes in the spatial perception of participants regarding, for instance, the perception of spatial relations between one-self and other objects, ultimately promoting the use of novel strategies for navigation^23^.

Future studies could further characterize the effects of using the Topo-Speech algorithm to augment visual spatial perception and extend the “visual field” from $180^\circ$ to $360^\circ$. For example, one can investigate the extent to which the new backward “visual” spatial sense would interfere with our natural visual sense by repeating the current experiment on participants without blindfolds^24^. Moreover, future studies should include the investigation of the neural mechanisms underlying this novel sensory experience. That is, where would this augmented backward space be represented in the brain? And specifically, would it be represented only in the auditory cortex or would it recruit the visual cortex as was repeatedly shown in cases of both blind and sighted SSD users^10,49,50^? As a matter of fact, the forward visual field is represented in the human brain through a very precise organization in early visual cortices (including the primary visual cortex (V1)), as well as in higher-order visual areas. This distinct organization is known as topographic mapping, or more specifically, retinotopic mapping^51–53^. This means that each part of the visual field is mapped to a specific region in the visual cortices, and closer parts in external space are processed in closer cortical regions. The field beyond 180° is not represented at all in the human visual system. Therefore, in case of augmented backward spatial processing, would such newly learned information be organized topographically? And if yes, where would such (novel?) maps emerge: in auditory regions, in parietal cortices (which are known to process spatial information), or perhaps in the visual cortices themselves?

Another aspect that could be addressed by future studies is the possible role of visual imagery in mediating, or perhaps facilitating forward and backward auditory spatial localization via the Topo-Speech. To answer this question, congenitally blind users can be tested in this task and their performance could be compared to sighted blindfolded adults while reaction times are collected (i.e., via a tablet for instance). If visual imagery does play a role in sighted performance, sighted participants may perform quicker than blind participants, as seminal evidence suggests that visual imagery has a facilitatory effect on perception^54–57^.

#### Implications for assistive technology and sensory substitution

Our results carry important implications for the realm of assistive technology and SSDs in particular. First, such results highlight that by using the Topo-Speech, it is possible to drastically reduce training time and yet be able to perform relatively complex tasks. This is particularly important considering that to use most SSD algorithms, long training programs are needed, which require continuous feedback from instructors^21,58^, thus making the learning process quite cumbersome. Furthermore, such training programs are highly cognitively-demanding, and users must invest hours of training to perform even very simple tasks with SSDs^10,11,13^. Both these aspects substantially limit SSD wide applicability in research, for instance as a promising tool to investigate the within-participants representations of the same spatial and perceptual processes carried out by different sensory modalities (e.g., vision and audition)^16–18^. Hence, by substantially reducing training time, the advantages we introduce to the SSD world with the Topo-Speech are enormous. Note that in addition to the aforementioned research venues, the Topo-Speech also paves the way for the systematic exploration of the multifaceted relations between language/semantics and more low-level sensory information^59,60^.

A natural follow-up to this study, which we are currently undertaking, is to test the efficacy of this algorithm with the blind and visually impaired population, namely the main target of SSD-like technologies. Such investigation will assess the extent to which the Topo-Speech algorithm is suitable to be used within visual rehabilitation settings, namely the classic and primary use of sensory-substitution algorithms^61^.

### Real world use: limitations and future directions

The current study should be considered as a proof-of-concept for the feasibility of the Topo-Speech algorithm to intuitively convey and augment “visual” spatial information via audition both in the frontal and backward spatial fields. Nonetheless, the results reported in this work, must be interpreted with respect to the current limitations of the algorithm, which ultimately hinder the Topo-speech real-world application. Please note that we intend to address such limitations in future studies and in future developments of the algorithm.

The first limitation of the Topo-Speech algorithm’s current version is that it uses pre-recorded stimuli, thus preventing the exploration of real-world environments, during which new objects continuously appear. We aim at solving this issue by implementing within the algorithm, an AI-based, machine-learning feature, for objects’ identification (similar to previously developed AI technologies^27^). Note that we chose in this proof-of-concept study not to implement this deep-learning feature and instead use only 2-syllables long words for two main reasons. First, we wanted to ensure words’ comprehension while maximizing the number of trials. Pilot studies showed that 2-syllables words allowed the highest words’ comprehension when each word lasted 50 ms (thus allowing trials to last 2 s in total—see Fig. 1a). Additionally, to exclude that potential differences in accuracy were caused by words’ length differences, we decided to present only 2-syllables words across the whole experiment. To make sure that Topo-Speech users will always understand the words they would hear, independently of their length, future developments of the Topo-Speech algorithm will include the addition of an object-to-speech speed converter, adapting the speed of the speech to the user’s preferences.

Additionally, in this work we only investigated the localization of one object at a time. Future studies should investigate whether accuracy of localization is influenced by the addition of multiple objects in the scene. Similarly, the grid of localization should be enlarged to include more positions than the 3 × 3 options available here. Both these changes will allow the Topo-Speech to more closely resemble real world-scenarios.

Moreover, The Topo-Speech does not currently provide any shape information. This is different from all previously discussed SSDs which convey detailed shape information, together with spatial locations information. Learning to interpret shape-related information is indeed the main focus of SSD training programs, ultimately determining their incredible length and related high cognitive demand^21^. The current version of the Topo-Speech forgoes the possibility of conveying exact shape information in favor of quickness of learning, thus aiming to make the algorithm more appealing both for everyday use^62^ as well as for research purposes. However, there might still be situations where detailed shape information is crucial for users, thus future developments of this algorithms will entail the option of also playing full shape information using typical SSD algorithms when needed by users.

Finally, for extending the Topo-Speech capacities and applicability, we plan on implementing new features in the Topo-Speech algorithm, such as conveying depth (z-axis positions) through volume manipulations (i.e., farther objects will be sonified with a lower volume) or conveying the x-axis positions through stereophonic sounds rather than through sound delays (the currently used approach).

We believe that together, these developments will provide Topo-Speech users with a more enriched perception of the 360° spatial environment, ultimately allowing to systematically investigate potential constraints related to sensory-specific inputs, such as vision, on the construction of spatial representations.

## Conclusions

In this paper we provided novel insights regarding the learning of new sensory skills during adulthood, i.e., augmented backward spatial localization. We presented a new SSD-inspired algorithm, the Topo-Speech, which conveys location and identity of objects via audition, namely two distinctive features of visual spatial perception. We first demonstrated the intuitiveness and efficacy of the algorithm for locating objects positioned in front of participants after minimal training (~ 10 min) (forward condition). Crucially, we showed in a new group of participants that the same visual-to-auditory mapping was comparably effective for localizing objects presented in the back of participants (backward condition), namely an area of space normally not accessible through vision. This, in turn, suggests that it is possible, at least to a certain extent, to augment or extend perceptions in the adult healthy population via our existing sensory systems, ultimately strengthening and extending initial evidence in this direction^4,23,24^. Given that our societies are becoming increasingly more technological and augmentation devices are quickly developing, systematically investigating this topic is becoming a priority. Such work would not only lead to advances in the understanding of the properties and constraints characterizing human sensory systems, but would also potentially guide the development of future technological applications.

## Supplementary Information

Supplementary Information.

## Data Availability

The datasets generated during and/or analyzed during the current study are available from the corresponding author on reasonable request.
