# Supplementary material for: Backward spatial perception can be augmented through a novel visual-to-auditory sensory substitution algorithm
Source: Sci Rep. 2021 Jun 7;11:11944. doi: 10.1038/s41598-021-88595-9 (PMC8184900; doi:10.1038/s41598-021-88595-9)
Supplement: Supplementary file 1 — Supplementary Information. [file 41598_2021_88595_MOESM1_ESM.pdf]

# Backward spatial perception can be augmented through a novel Visual-to-Auditory sensory substitution algorithm

**Ophir Netzer*[1], Benedetta Heimler*[2,3,4], Amir Shur[1], Tomer Behor[1], Amir Amedi[2,3]**

1- The Cognitive Science Program, The Hebrew University of Jerusalem, Jerusalem, Israel

2- The Baruch Ivcher Institute For Brain, Cognition & Technology, The Baruch Ivcher School of Psychology, Interdisciplinary Center Herzliya, Herzeliya, Israel

3- Department of Medical Neurobiology, Hebrew University of Jerusalem, Hadassah Ein-Kerem, Jerusalem, Israel

4- Center of Advanced Technologies in Rehabilitation (CATR), Sheba Medical Center, Ramat Gan, Israel

*Equal contributions

**Supplementary material**

**Preliminary experiment - *Words selection***

The aim of this preliminary experiment was to exclude from the Topo-Speech experimental pool words that had a spatial bias in the y-axis (high vs. low), as they might have an influence on participants responses (e.g., "carpet"- may be associated with the lower part of the space; "cloud"- may be associated with the upper part of the space).

**Methods**

B. Equipment

**Experimental set-up.** An online questionnaire was constructed for the purpose of this experiment using the Qualtrics platform (https://www.qualtrics.com/).

C. Experimental design and procedure

Participants sat in front of a computer screen. In each trial, participants saw one word in the center of the screen with a sliding bar beneath it. On the right end of the bar the word "low" was written, while the word "high" was written on its left end in Hebrew (Supplementary Figure S1). Participants were instructed to use the marker on the sliding bar to indicate their perception of a spatial vertical bias for each given word (in Supplementary Figure S1 the word is "shaon" which means "clock"). At the beginning of each trial, the marker was placed in the middle of the sliding bar and participants needed to slide it to the left or to the right if they felt the object is naturally located in the higher or lower part of space. Also, they could choose the strength of a possible vertical spatial bias (e.g., the closer to the end in any of the two direction of the bar, the stronger

the bias). If they did not feel that the object had any particular height attribution, they were instructed to leave the marker in the middle of the bar (its default location). The duration of the experiment circled around 6 minutes. Participants were encouraged to respond as quickly and as intuitively as possible.

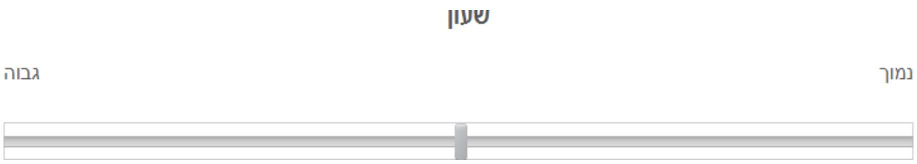


**Supplementary Figure S1- An example of an experimental trial.**

**Results**

Even though participants saw a continuous bar on the screen with the words “high” and “low” at the edges (Supplementary Figure S1), for the purpose of the analysis, we assigned hidden values to the bar ranging from 0 to 100. The score 0 represented the “low” edge of the scale; the score 100 represented the “high” edge of the scale; the score 50 represented the middle (or - no specific height attribution). To test for any spatial bias in the y-axis, we performed a one-sample two-tailed t-test on each word comparing the responses of all participants to that specific word against 50, i.e., the value corresponding to “no specific spatial bias”. A p-value $<0.05$ indicates that there is a spatial bias to the object (either high or low). Twenty-six out of the total of sixty words were found to have significant spatial bias in one of the two directions (Supplementary Table S1). Specifically, the words resulting to have a spatial bias were: clock, hat, chair, bag, sock, shoe, carrot, scarf, wand, eggplant, lightbulb, wheel, crate, balloon, flower, flag, newspaper, flip -flop, planter, charger, earring, boot, tree-branch, oyster, can, hanger (all p-values $< 0.05$). All of the above words

were removed from the pool of words used for the Topo-Speech experiment to follow, even though some of these words were used during the related training (experiments 2).

| Word in Hebrew | Translation to English | Mean score | Standard deviation | t-value | p-value | Used in |
|---|---|---|---|---|---|---|
| כדור | Ball | 34.55 | 29.32 | -1.748 | 0.111 | Experiment |
| בקבוק | Bottle | 48.18 | 17.05 | -0.354 | 0.731 | Experiment |
| קופסה | Box | 38.45 | 17.4 | -2.2 | 0.052 | Experiment |
| כפתור | Button | 47 | 7.28 | -1.367 | 0.202 | Experiment |
| מחשב | Computer | 49.45 | 18.05 | -0.1 | 0.922 | Experiment |
| חליל | Flute | 60.09 | 20.05 | 1.669 | 0.126 | Experiment |
| מזלג | Fork | 44.27 | 18.37 | -1.034 | 0.325 | Experiment |
| כפפה | Glove | 48.91 | 13.7 | -0.264 | 0.797 | Experiment |
| פטיש | Hammer | 46.27 | 18.97 | -0.652 | 0.529 | Experiment |
| קומקום | Kettle | 47.45 | 14.42 | -0.585 | 0.571 | Experiment |
| סכין | Knife | 43.64 | 14.13 | -1.494 | 0.166 | Experiment |
| עלה | Leaf | 70.18 | 30.7 | 2.18 | 0.054 | Experiment |
| לימון | Lemon | 50.18 | 25.73 | 0.023 | 0.982 | Experiment |
| מכתב | Letter | 48.18 | 15.85 | -0.38 | 0.712 | Experiment |
| מצית | Lighter | 41.18 | 14.82 | -1.973 | 0.077 | Experiment |
| מנעול | Lock | 43.64 | 18.89 | -1.117 | 0.29 | Experiment |
| ספל | Mug | 39 | 17.03 | -2.142 | 0.058 | Experiment |
| פנקס | Notepad | 43.09 | 11.79 | -1.944 | 0.08 | Experiment |
| תפוז | Orange | 55.27 | 18.85 | 0.928 | 0.375 | Experiment |
| מכחול | Paintbrush | 45.45 | 15.5 | -0.973 | 0.354 | Experiment |
| מחבת | Pan | 43.82 | 15.08 | -1.36 | 0.204 | Experiment |
| קלמר | Pencil box | 48 | 9.51 | -0.698 | 0.501 | Experiment |
| סיכה | Pin | 57.09 | 13.6 | 1.729 | 0.115 | Experiment |
| קלסר | Ring binder | 47.82 | 17.37 | -0.417 | 0.686 | Experiment |
| חבל | Rope | 48.55 | 16.34 | -0.295 | 0.774 | Experiment |
| סרגל | Ruler | 47 | 17.02 | -0.585 | 0.572 | Experiment |
| חולצה | Shirt | 58.73 | 16.08 | 1.801 | 0.102 | Experiment |
| כפית | Spoon | 44.55 | 14.89 | -1.215 | 0.252 | Experiment |
| שדכן | Stapler | 45.09 | 14.27 | -1.141 | 0.28 | Experiment |
| ארנק | Wallet | 45.91 | 15.1 | -0.898 | 0.39 | Experiment |
| בלון | Balloon | 82.18 | 21.84 | 4.886 | 0.001 | Training |
| מגף | Boot | 12.18 | 18.13 | -6.918 | 0.0001 | Training |
| גזר | Carrot | 33.82 | 20.99 | -2.556 | 0.029 | Training |
| כיסא | Chair | 32.18 | 22.32 | -2.648 | 0.024 | Training |
| ארגז | Crate | 24.91 | 23.33 | -3.568 | 0.005 | Training |

| | | | | | | |
|---|---|---|---|---|---|---|
| בובה | Doll | 51.82 | 17.44 | 0.346 | 0.737 | Training |
| עגיל | Earring | 65.91 | 18.79 | 2.809 | 0.019 | Training |
| ביצה | Egg | 45.73 | 21.6 | -0.656 | 0.527 | Training |
| דגל | Flag | 82.36 | 18.53 | 5.792 | 0.0002 | Training |
| כפכף | Flip flop | 9.18 | 16.73 | -8.091 | 0.0001 | Training |
| פרח | Flower | 29.45 | 19.79 | -3.443 | 0.006 | Training |
| כובע | Hat | 78.36 | 17.5 | 5.375 | 0.0003 | Training |
| עיתון | Newspaper | 29.09 | 21.31 | -3.254 | 0.009 | Training |
| קנקן | Pitcher | 45.82 | 17.16 | -0.808 | 0.438 | Training |
| עציץ | Planter | 29.73 | 16.53 | -4.068 | 0.002 | Training |
| נעל | Shoe | 11.09 | 19.59 | -6.586 | 0.0001 | Training |
| סבון | Soap | 39.64 | 19.77 | -1.739 | 0.113 | Training |
| גרב | Sock | 16.45 | 21.9 | -5.08 | 0.0005 | Training |
| ענף | Tree branch | 75.91 | 20.69 | 4.153 | 0.002 | Training |
| שרביט | Wand | 66.55 | 18.69 | 2.936 | 0.015 | Training |
| שקית | Bag | 37 | 15.55 | -2.773 | 0.02 | Discarded |
| פחית | Can | 36.27 | 15.8 | -2.882 | 0.016 | Discarded |
| מטען | Charger | 32.36 | 24.23 | -2.414 | 0.036 | Discarded |
| שעון | Clock | 61.55 | 16.78 | 2.282 | 0.046 | Discarded |
| חציל | Eggplant | 30.18 | 18.83 | -3.491 | 0.006 | Discarded |
| קולב | Hanger | 65.18 | 11.69 | 4.309 | 0.002 | Discarded |
| נורה | Light bulb | 85.73 | 17.05 | 6.948 | 0.0001 | Discarded |
| צדפה | Oyster | 21.82 | 25.06 | -3.729 | 0.004 | Discarded |
| צעיף | Scarf | 71.45 | 15.19 | 4.685 | 0.001 | Discarded |
| גלגל | Wheel | 13.91 | 20.09 | -5.958 | 0.0001 | Discarded |

**Supplementary Table S1- The list of words tested.** Column 1- the stimulus presented (the word in Hebrew). Column 2- the translation of the word to English. Column 3- the mean score of the word across subjects (0-lowest edge; 100- highest edge). Column 4- standard deviation of the score across subjects. Column 5- t-value for each word in the one-sample two-tailed t-test (positive score represents tendency for high spatial bias; negative score represents a tendency for low spatial bias). Column 6- p-value for each word in the t-test. Column 7- the part of the experiment the word was used in. The possibilities are: used in training, used in the experiment or discarded (not used in any part).

**Evaluation of the data distribution-** ***estimating density plots***

Prior to performing any statistical analyses, we evaluated the distributions of the data tested in each experimental condition, to choose the appropriate statistical analyses (i.e., if the data meets the assumptions of parametric testing). Supplementary Figure S2 presents the density plots for the individual average success rates of participants in the forward and backward conditions separately, to visualize the underlying probability distribution of the data (for specified individual average success rates please see the Results section in the main text, specifically- Table 1 for forward condition; Table 2 for backward condition).

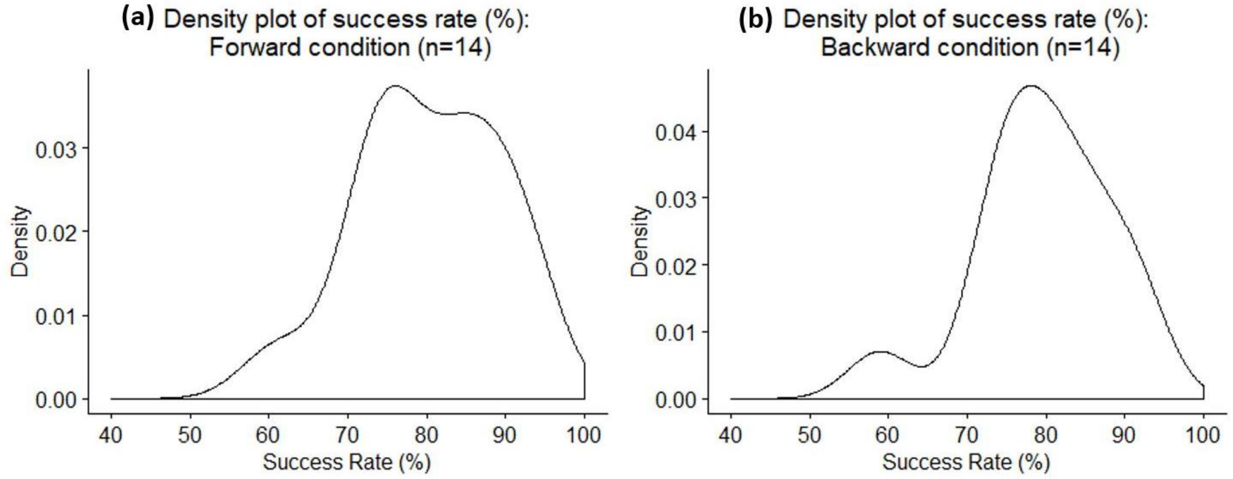


**Supplementary Figure S2- Density plots of individual average success rates. (a)** Blindfolded sighted participants in the forward vision condition (n=14). **(b)** Blindfolded sighted participants in the backward vision condition (n=14). Both density plots for the data in each condition, suggest that both the evaluated distributions tended to be negatively skewed.
